# Toxic Effects of Methanol among Illegally Dispatched Workers at Aluminum CNC Cutting Process in Small-Scale, Third-Tier Subcontractor Factories of Smartphone Manufacturers in the Republic of Korea

**DOI:** 10.3390/ijerph15071332

**Published:** 2018-06-25

**Authors:** Chung Won Kang, Hyunjoo Kim, Kyongsok Shin, Jia Ryu, Kyunghee Jung-Choi, Key Hwan Lim, Jin-Ha Kim

**Affiliations:** 1 Department of Occupational and Environmental Medicine, Ewha Womans University Mokdong Hospital, Seoul 07985, Korea; scmfer@hanmail.net; 2 Center for Bio-Environmental Science, Seegene Medical Foundation, Seoul 04805, Korea; i.medicine@hotmail.com; 3 Department of Occupational and Environmental Medicine, College of Medicine, Ewha Womans University, Seoul 07985, Korea; jiyajiya000@gmail.com (J.R.); jungchoikh@gmail.com (K.J.-C.); 4 Department of Ophthalmology, College of Medicine, Ewha Womans University, Seoul 07985, Korea; limkh@ewha.ac.kr (K.H.L.); jinhakim11@gmail.com (J.-H.K.)

**Keywords:** methanol exposure, toxic effects, subcontractor manufacturing, dispatched workers, visual defect, neurobehavioral function

## Abstract

An outbreak of occupational methanol poisoning occurred in small-scale, third-tier factories of large-scale smartphone manufacturers in the Republic of Korea in 2016. To investigate the working environment and the health effects of methanol exposure among co-workers in the methanol poisoning cases, we performed a cross-sectional study on 155 workers at five aluminum Computerized Numerical Control (CNC) cutting factories. Gas chromatography measured air and urinary methanol concentration. In the medical examination, symptom surveys, ophthalmological examinations, and neurobehavioral tests were done. Multiple logistic regression analyses controlling for age and sex were conducted to reveal the association of employment duration with symptoms. Air concentrations of methanol in factory A and E ranged from 228.5 to 2220.0 ppm. Mean urinary methanol concentrations of the workers in each factory were from 3.5 mg/L up to 91.2 mg/L. The odds ratios for symptoms of deteriorating vision and central nervous system (CNS) increased according to the employment duration after adjusting for age and sex. Four cases with an injured optic nerve and two cases with decreased neurobehavioral function were founded among co-workers of the victims. This study showed that the methanol exposure under poor environmental control not only produces eye and CNS symptoms but also affects neurobehavioral function and the optic nerve. The role of subcontracting production and dispatched work under poor environmental control was discussed.

## 1. Introduction

Methanol, widely used in various processes for a long period after the 1900s, is a light, volatile, colorless, flammable liquid with a distinctive odor very similar to that of ethanol. The first case of occupational methanol poisoning, reported in 1901, was of a man who became blind after periodic exposure to varnish dissolved in methanol and from the use of methanol to clean his face and arms over a period of three years [1]. Greenburg et al. performed the first epidemiologic study. They studied 19 workers employed in the manufacture of “fused collars”. Concentrations of methanol in the workroom were measured to be 29–33 mg/m^3^, and a “strong odor” of solvent was perceptible. The shortest period that any of these workers had spent fusing collars was nine months, and the longest was two years. No central nervous system(CNS) or visual anomalies in any of these workers were reported [2]. 

Chronic and acute exposures to methanol vapors have been studied at or near the limits of allowable methanol vapor exposure. According to an American Conference of Governmental Industrial Hygienists (ACGIH) report, there were several known instances of occupational methanol poisoning and the related investigations until 1960s were as follows: A study indicated severe, recurrent headaches in workers exposed to methanol at concentrations between 200 and 375 ppm [3]. Another study of the wood heel industry found average methanol vapor concentrations ranging from 160 to 780 ppm, with no definitive evidence of injury to the exposed workers [4]. Visual disturbances, including dilated unreactive pupils and dim vision, were reported from airborne methanol concentrations of 1200–8300 ppm [5]. One case of chronic methanol poisoning resulted from exposure at 1200–8000 ppm for four years [6]. The current occupational limits of 200 ppm in air averaged over 8- or 10-h days and 40-h weeks were established in 1948. Two hundred and fifty ppm averaged over 15 min was established as the short-term exposure limit in 1976, and 6000 ppm for the immediately dangerous concentration to life and health was set in 1994 [7]. A more recent epidemiological study on the effects of occupational methanol exposure was conducted in 1984. The National Institute for Occupational Safety & Health (NIOSH) published a study of teacher aides who worked at or near spirit duplicators that used a 99% methanol duplicator fluid for about three years. A health questionnaire survey was conducted and suggested that chronic effects may occur when methanol concentrations exceed the threshold limit value of 260 mg/m^3^. However, additional clinical investigations did not proceed to define the effects further [8].

Occupational methanol poisoning seems to disappear into the mists of history with the establishment of these exposure limit values for methanol as well as the settlement of regulations on occupational health and safety, especially in developed countries. Most of the methanol poisonings recently reported in the articles were due to nonoccupational exposure to methanol, mainly by ingestion or intentional inhalation, that is, abuse [9]. Only a few accidental poisonings due to occupational inhalational or dermal exposure were reported among nonmanual workers such as laboratory workers [10] and consultants [11]. However, an outbreak of occupational methanol poisoning occurred in the manufacturing industry in the Republic of Korea in 2016. Two cases involved people who worked for several months at an aluminum Computerized Numerical Control (CNC) cutting process in a small-scale, third-tier subcontractor factory for large-scale smartphone manufacturing companies [12]. An acute poisoning case of a person who had worked for just several days at the same process in another company was found a week after the first case was reported. The fourth case was reported one month later, near the district where the former cases had occurred [13]. Furthermore, cases of another two workers who had been poisoned at the above factories were reported in September 2016 [14]. We performed special health examinations in order to protect their co-workers from methanol exposure and to reveal the cause of this outbreak of occupational poisonings according to the administrative order to the employers by the Ministry of Labor and Employment.

This study aimed to investigate the working environment of the aluminum CNC cutting process in small-scale, third-tier subcontractor factories of smartphone manufacturers and the health effects of methanol exposure among co-workers of the poisoning cases.

## 2. Materials and Methods

### 2.1. Study Population

This retrospective observational study was conducted on 155 workers who had worked at five small-scale factories. We performed check-ups on the workers with special health examinations just after the outbreak of the occupational methanol poisonings. They worked at the aluminum CNC cutting process in five small-scale factories named A–E according to the order of the investigation, and these factories were located in Bu-cheon city, Kyoung-gi province, the Republic of Korea. Factory A, where two cases of subchronic poisoning occurred, and factories B, C, and D supplied aluminum buttons to the same parent company which was also a second-tier subcontractor of large-scale smartphone manufacturing companies. Factory E, where an acute poisoning case was found, did work for another second-tier subcontractor of the same smartphone companies. All of the employees who worked at the factory during the day when the hospital staff visited the factories to collect urine samples participated.

### 2.2. Exposure Assessment

From 22–26 January 2016, environmental samplings and analyses were conducted by labor inspectors affiliated with the Bu-cheon district office of the Korea Labor and Employment office.

Factories A and E used methyl alcohol. Since factories B, C, and D changed the cooling agent from methanol to ethanol just before the environmental sampling after hearing that methanol poisoning had occurred at the same process, ethanol levels were measured in these factories. The workplace air concentration of methanol and methanol-containing proportions in bulk samples were analyzed using gas chromatography by the laboratory of the Korea Occupational Safety and Health Agency (KOSHA). The results were delivered to the occupational physician of the hospital, who performed the special health examination. From 25–29 January 2016, the work-through survey on each factory and interviews with the employers or managers by an occupational physician were conducted and the urine from 146 workers for biological monitoring was sampled. Most of the urine sampled was not taken at the end of the shift, except for day-shift workers in factory E. The sampling had to be conducted before the shut-down of factories A and E. The others had already stopped using methyl alcohol. Urinary methanol levels were measured by the Seegene Medical Foundation, Seoul, Korea. Gas chromatography was performed on an Agilent 7890 series gas chromatograph (Agilent Technologies, Santa Clara, CA, USA) with a flame ionization detector. The system was equipped with a CombiPaL Headspace Sampler. A 60 m × 0.250 mm × 1.4 um DB-624 (Agilent Technologies, Santa Clara, CA, USA) column was used. To 10 mL headspace vials, 3 mL of either calibration, urine samples were added. Vials were capped quickly to avoid loss of analytes by volatilization and were transferred to the autosampler.

### 2.3. Special Health Examination

The special health examination was conducted at the department of occupational and environmental medicine of a university hospital from 26 January to 12 February 2016. The participants were 155 workers, including 146 workers whose urine had been sampled before the special health examination and 9 workers who had not worked at that time when the hospital staff visited there. Two workers were absent on the day of the examination because of illnesses related to methanol exposure. The other seven workers did not go to work on the night of the day of the examination because they had heard that the factories were shut down. The examination included inquiries regarding work history, symptoms, ophthalmological examinations, and neurobehavioral function. The structured symptom questionnaire for the special health examination based on KOSHA guidelines was used to systematically collect the data on the workers’ symptoms. In this questionnaire, methanol-exposure-related symptoms were regarded as follows: (i) My eyesight is worse than before; (ii) I feel headaches; (iii) I feel dizzy; (iv) I have become more forgetful; (v) I am anxious and restless; (vi) My head feels numb or I feel as though I am drunk; and (vii) I find it hard to concentrate. All workers were checked for visual acuity and were subjected to a color vision test by Han’s method and fundus photography. Thirty-six workers who complained about worsened eyesight or showed abnormal findings of the fundus photography had their visual field examined (30-2 SITA-standard strategy, Humphrey visual field analyzer HFA750i; Carl Zeiss Meditec Inc., Dublin, CA, USA). Simple reaction and symbol digit substitution was tested on 90 workers by a Korean computerized neurobehavioral test system [15]. The reason why all workers could not undergo the neurobehavioral test was that the workers, who visited the hospital just after 12 h of night-shift work, could not meet the prerequisites for testing. That is, this test cannot obtain accurate results when the subject is very tired or sleepy. The neurobehavioral test was conducted on all workers except for one production manager who had refused the test because of nonexposure in the factories A and E where the poisoning cases had occurred. Seventeen of 44 workers in factory B and 13 of 37 workers in factory C could be tested. All of the 13 workers in factory D did not need to be tested.

### 2.4. Statistical Analysis

IBM SPSS Statistics for Windows, Version 22.0. Armonk, NY: IBM Corp. was used. The descriptive statistics were calculated for explaining the characteristics of the study population and the factories. Chi-squared tests were conducted to reveal the significant exposure variables associated with symptoms of deteriorating vision and the central nervous system. Only the employment duration had shown statistically significant associations with the symptoms (*p* < 0.05). The other variables related to methanol exposure, such as factory, type of employment, and process did not. Therefore, workers who had worked less than one month were defined as the low-exposure group, which was used as an internal control group. Multiple logistic regression analyses controlling for age and sex were conducted to reveal the association of employment duration with symptoms related to methanol exposure. 

### 2.5. Ethical Consideration

The study was approved by the institutional review board of Ewha Womans University Mok-dong Hospital. (Approval No. 2016-05-048-002).

## 3. Results

More male workers (69.7%) than female workers (30.3%) worked at the five small-scale factories. The most common age group was the twenties (49.7%), followed by thirties (32.3%) and forties (18.1%). The percentage of alcohol drinkers was 51.0%. The number of the workers was 29 in factory A, 44 (28.4%) in factory B, 37 (23.9%) in factory C, 13 (8.4%) in factory D, and 32 (20.6%) in factory E. They worked at the processes of aluminum CNC cutting machine operation (60.0%), measuring the size of aluminum buttons (16.1%), visual inspection of the aluminum buttons (7.7%), and production management (16.1%). Their duration of employment was relatively short: 19.4% for less than one month, 38.1% for from one to three months, and 42.6% for more than three months. Eighty percent of the study population were illegally dispatched workers, and only 20% of those were permanent workers. The proportion of migrant workers was 41.3%. The symptom prevalence of deteriorating vision and central nervous system was 29.7% and 31.6%, respectively. 3.9% of workers showed abnormal clinical findings, which were abnormal ophthalmological findings or abnormal results of the neurobehavioral test (Table 1).

Regarding the characteristics of the factories, the numbers of the involved dispatch agencies ranged from two to four in each factory, and the proportions of dispatched workers were from 62.2 to 100%. Those of migrant workers were from 3.4 to 69.2%. The number of the aluminum CNC cutting machines in each factory varied from 29 to 66. All of the machines were open mode, from which methanol easily vaporized and disseminated in factory E. Almost all of the machines, 24 of 29, were open mode in factory A. There were more open than closed machines in the factories other than factories A and E. Environmental samplings were conducted during routine operations in factories A and E, which used 99.9% methanol as the cooling agent for aluminum CNC cutting machines. The air concentration of methanol ranged from 1030.1 to 2220.0 ppm in factory A and from 228.5 to 417.7 ppm in factory E. The ethyl alcohol levels of factories B, C, and D were measured, and their levels ranged from 22.5 to 128.7 ppm. Mean urine concentrations of methanol in factories C and D were slightly higher than the reference value of 2.0 mg/L. Factory B showed 8.6 ± 16.2 mg/L of methanol in urine because their workers used still 30% methanol-containing coolant. In factory A, a mean of 7.9 ± 7.1 mg/L of methanol in urine was detected, although their urine was sampled one day after shutting down. A mean of 91.2 ± 85.8 mg/L of methanol in urine was found among workers in factory E. In some factories, the range of urinary methanol concentrations was very wide and the standard deviation was larger than the mean values. For example, the maximum concentration of urinary methanol in factory B was 107.2 mg/L. There was no statistical correspondence between exposure level in the workplace and urinary methanol concentrations since the environmental measurements and urine tests were conducted at different times and conditions at each workplace (Table 2).

Workers with deteriorating vision were 29.7% of those tested. The odds ratios for deteriorating vision among workers with one to three months of employment and those with more than three months were 2.242 (95% confidence interval (C.I.), 0.345–4.044) and 3.487 (95% C.I., 1.164–10.449), respectively, compared to workers with less than one month of employment in multiple logistic regression analyses controlling for age and sex. Workers with CNS symptoms were 31.6%. The odds ratios for CNS symptoms were 2.775 (95% C.I., 0.808–9.528) and 4.611 (95% C.I., 1.377–15.440), respectively. The odds ratios for the symptoms among workers with 13 months of employment compared to those with less than one month were not statistically significant. They were significant only among those with more than three months (Table 3).

The results of visual acuity and color vision tests did not suggest methanol-induced eye problems. However, we found four cases with visual field defects and two cases with abnormal neurobehavioral tests. Two cases with optic nerve abnormality detected by ophthalmoscopy had worked in factories B and C. Another two cases with decreased neurobehavioral function were also found in factories B and C (Table 4).

## 4. Discussion

In this study, we found a statistically significant association of eye and CNS symptoms with the employment duration among co-workers of methanol poisoning cases. Furthermore, six cases with abnormal findings of neurobehavioral function or optic nerve dysfunction were detected.

Symptom prevalence of deteriorating vision increased according to the duration of employment. This result is consistent with the former studies mentioned in the introduction. We found four cases of subclinical visual defects. One case was exposed to methanol at the concentration of 2220 ppm only for several days. The other three cases were supposed to be exposed to relatively low levels of methanol for several months. However, there was no additional case in factory E, irrespective of their workers’ 6.2 times higher levels of urinary methanol compared to the occupational exposure limit of the biological exposure index (15 mg/L). This may be because of their short exposure period. This ocular toxicity appears to be caused by formic acid directly, the metabolite of methanol, and not by the metabolic acidosis that accompanies its accumulation. Acidosis can increase toxicity further by enabling greater diffusion of formic acid into cells. Undissociated formic acid specifically targets the optic disc and retrolaminar section of the optic nerve, causing optic disc edema, the breakdown of the myelin sheaths, and optic nerve lesions [16].

Regarding the effects of methanol on the central nervous system, there were significant differences in symptoms according to the duration of employment in this study. This finding suggests that the cumulative levels of exposure played an important role in methanol toxicity. There were two cases with abnormal results of the neurobehavioral tests in this study, which may reflect that exposure to methanol produces an adverse effect on the attention and the perceptual-motor speed of the workers. The epidemiological studies published before these neurobehavioral tests had developed described CNS symptoms such as headaches and difficulty in concentration. In later studies, with the use of various tools for neurobehavioral tests, typically decreased neurobehavioral function was demonstrated in workers exposed to organic solvents [17]. Methanol, like other organic solvents, can be expected to harm white matter in addition to its specific damage to the basal ganglia.

The ocular and CNS effects of methanol mentioned above were observed at relatively low air concentrations of methanol compared to the former studies. Moreover, it is known that methanol toxicity is lower in inhalational exposure than in cases of ingestion [18], since pharmacokinetic theory indicates that the peak level of methanol after inhalation does not reflect the same body burden of formate as an equivalent level after oral exposure [16]. In this study, it is interesting that there was a gap between urinary and air concentrations of methanol, which is supposed to result from the considerable amount of dermal exposure. According to work through and interviews by an occupational physician, dermal exposure in these CNC cutting factories was possible when working without proper protective clothes and gloves, especially during the task of dividing coolant, which is usually performed in turns. Two workers with subchronic poisoning did not wear any protective gloves, clothes, or respirators since those were not provided [12]. Exposure of one hand to liquid methanol for only two minutes would lead to the absorption of as much methanol as would be taken up by the lungs from an eight-hour exposure to an air concentration of 50 mg/m^3^ [19]. Therefore, the primary route of exposure to methanol seems to be inhalational, but dermal exposure may also play an important role in increasing the body methanol exposure level.

Finally, what we would like to point out is that workers in the small-scale, third-tier subcontractor factories of large-scale smartphone manufacturers were exposed to methanol at a maximum air concentration of about 10-fold higher compared to the occupational exposure limit. It is hard for this to happen in developed countries because of established occupational safety and health systems. However, it is possible because subcontracting within the supply chain for electronics manufacturer is known to pursue maximized profits in the electronics industry, which depends on the systems of “cost down” and “flexibility” [20]. Large companies can catch two rabbits: lowering costs and outsourcing risk through subcontracted production. Subcontractors cannot manage the “risk” because they must keep costs as low as possible to meet the delivery price. Their workers had to use methanol as a coolant instead of ethanol, which is less toxic and does not produce optic neuropathy, because the cost of ethanol is four-fold more expensive compared to that of methanol. Furthermore, they used cheap open-mode machines instead of expensive closed-mode machines, which can prevent from methanol vapor from disseminating [14]. In particular, these factories were run without a holiday ahead of the release of new products during the period of methanol poisonings in 2016.

Dispatched labor is prevalent because it is a very effective strategy for ensuring flexibility of labor in the electronics industry, although it is prohibited by the Korean Labor Standard Law. This phenomenon was accelerated in the case of the multilayered subcontracting. As seen in this study, 14 dispatch agencies were involved in the five small-scale factories to provide a smooth workforce to prepare for fluctuating orders and to meet the lowest supply price for the large-scale smartphone manufacturers. In this study, the proportions of dispatched workers in each factory were from 62.2% to 100%. The symptom prevalence or the rate of abnormal clinical findings did not show a statistically significant difference according to the type of employment. However, the dispatched work could contribute to increasing the risk of the toxic effects of methanol as follows: First, a company employing dispatched workers can avoid regulations by the Industrial Safety and Health Act of Korea because it is not required to appoint a health manager as a small business with fewer than 50 workers. Second, employers who give actual work instructions do not have an obligation to protect the health of dispatched workers. Employers of staffing agencies that hire them are formally responsible for their health protection, but they cannot protect their health because they do not manage the real working environment of their workers. Third, the dispatched workers cannot be provided with proper information on workplace hazards and personal protective equipment from both types of employers.

The limitations of this study are as follows. First, it is possible that this study population did not include all the workers who were exposed to methanol at the five factories. Although this health examination was conducted by an administrative order based on the occupational health and safety law, some employers and workers would not comply. Second, we have not shown statistical associations between methanol concentrations and symptoms of eye and CNS dysfuction, since environmental measurements and urine tests were conducted at the different times and conditions at each workplace. This investigation was made urgently according to the occurrence of acute methanol poisoning. The labor inspector first visited the workplace without notice and measured the work environment. Then, when an administrative order for a special health examination came down, the hospital staff could visit the workplace and collect the urine. Therefore, the methanol exposure level of the workplace cannot be statistically compared with the measured values themselves. Third, the cases with abnormal clinical findings could be underreported since workers only who complained of eye symptoms were referred to the ophthalmologist and some workers could not receive neurobehavioral tests. These are not limitations that authors can overcome.

Despite these limitations, it is worth reporting that we have identified the toxic effects of high concentrations of methanol, which rarely occurred in modern industrialized countries. Furthermore, this study has significance in that it shows that subcontracted production and dispatched work constitute a blind spot in managing hazardous factors in the workplace and can lead to poisoning by high concentrations of exposure to chemicals.

## 5. Conclusions

In short, this study showed that an about 10-fold higher level of methanol by inhalational and dermal exposure for several days to months can produce toxic effects on the eyes and central nervous system. Methanol itself would not be severely toxic if the work environment had been properly controlled. “Outsourcing risk” is toxic because subcontractor manufacturing and dispatched labor are faced with hazardous tasks without any protective measures. Regulations such as the prohibition of dispatched labor and subcontractor manufacturing for hazardous work should be kept to prevent such occupational poisonings from occurring again.

## Figures and Tables

**Table 1:** Characteristics of co-workers of methanol poisoning cases (*n* = 155). Unit: %.

Characteristics		Frequency	(%)
Sex	Male	108	(69.7)
	Female	47	(30.3)
Age	<29	77	(49.7)
	30–39	50	(32.3)
	40 ≤	28	(18.1)
Alcohol drinking	no	76	(49.0)
	yes	79	(51.0)
Factory	A	29	(18.7)
	B	44	(28.4)
	C	37	(23.9)
	D	13	(8.4)
	E	32	(20.6)
Migrant workers	no	91	(58.7)
	yes	64	(41.3)
Type of employment	permanent	31	(20.0)
	dispatched	124	(80.0)
Process	aluminum CNC cutting machine operating	93	(60.0)
	measuring the size of aluminum buttons	25	(16.1)
	visual inspection for aluminum buttons	12	(7.7)
	production management	25	(16.1)
Duration of employment	<1 month	30	(19.4)
	1–3 months	59	(38.1)
	>3 months	66	(42.6)
Deteriorating vision	no	109	(70.3)
	yes	46	(29.7)
CNS symptoms	no	106	(68.4)
	yes	49	(31.6)
Abnormal clinical findings	no	149	(96.1)
	yes	6	(3.9)

**Table 2:** Characteristics of the working conditions and the exposure to organic solvents in the five factories.

Factory	A	B	C	D	E
Working conditions					
Number of involved dispatch agency	2	4	3	3	2
Proportions of dispatched workers (%)	69.0	83.7	62.2	92.3	100
Proportions of migrant workers (%)	3.4	40.9	45.9	69.2	59.4
Organic solvent exposure					
Number of CNC cutting machines (open mode/total)	24/29	17/49	18/54	3/25	66/66
Methyl alcohol in bulk samples (%)	99.9	30.0	0	0	99.9
Air concentration (ppm)	Methyl alcohol	Ethyl alcohol	Ethyl alcohol	Ethyl alcohol	Methyl alcohol
Aluminum CNC cutting machine 1	1656.3	93.7	22.5	94.6	347.9
Aluminum CNC cutting machine 2	2220.0	18.9	34.4	128.7	228.5
Aluminum CNC cutting machine 3	1030.1		23.0		417.7
Aluminum CNC cutting machine 4					252.9
Places for measuring the size of aluminum buttons 1	2052.2	94.0			231.1
Places for measuring the size of aluminum buttons 2	1103.5				
Number of the urine sample	24	44	37	11	30
Urinary methanol concentration (M(SD), mg/L)	7.9 (7.1)	8.6 (16.2)	3.5 (3.0)	4.7 (6.8)	91.2 (85.8)

**Table 3:** Association of employment duration with methanol-exposure-related symptoms: multiple logistic regression analysis.

Duration of Employment	*n*	Case	Crude OR (95% C.I.)	Adjusted OR (95% C.I.) ^1^
Deteriorating vision	155	46		
<1 month	29	5	1	1
1–3 months	58	12	1.252 (0.395–3.971)	2. 242 (0.354–4.014)
>3 months	68	29	3.569 (1.216–10.476)	3.487 (1.164–10.449)
CNS Symptoms	155	49		
<1 month	29	4	1	1
1–3 months	58	18	2.250 (0.742–6.825)	2. 775 (0.808–9.528)
>3 months	68	27	3.537 (1.211–10.326)	4.611 (1.377–15.440)

^1^ Adjusted for age and sex.

**Table 4:** Abnormal clinical findings by the special health examination.

	Age/Sex	Factory	Entry Date	Symptoms	Ophthalmological Findings	Neurobehavioral Test (Delayed > 90th Percentile)
1	M/26	C	14 February 2014	Headaches, dizziness, difficulty in memory and concentration	Visual field defect Abnormal optic nerve	Normal
2	F/29	B	2 October 2015	None	Visual field defect Abnormal optic nerve	Delayed Simple reaction time
3	F/36	C	26 November 2015	None	Visual field defect	N/A
4	M/20	A	15 January 2016	Sickness absence due to eye, skin, cardiopulmonary, CNS symptoms	Visual field defect	Normal
5	M/33	C	6 January 2016	None	Not applicable	Delayed simple reaction time and symbol digit substitution time
6	M/50	B	15 January 2014	None	Not applicable	Delayed simple reaction time and symbol digit substitution time

## References

[1] Wood C.A., Buller F. (1904). Poisoning by wood alcohol. Cases of death and blindness from columbian spirits and other methylated preparations. J. Am. Med. Assoc..

[2] Greenburg L., Mayers M.R., Goldwater L.J., Burke W. (1938). Health hazards in the manufacture of ‘fused collars’ II Exposure to acetone-methanol. J. Ind. Hyg. Toxicol..

[3] Kingsley W.H., Hirsch F.C. (1995). Toxicologic Considerations in Direct Process Spirit Duplicating Machines. Compen. Med..

[4] Massachusetts Division of Occupational Hygiene (1937). Health Hazards of Wood Heel Covering. Quoted in American Conference of Governmental Industrial Hygienists (ACGIH) Documentation for methanol. Documentation of the Threshold Limit Values and Biological Exposure Indices.

[5] Humperdinck K. (1941). On the Problem of Chronic Intoxication with Methanol Vapors. Arch. Gewerbepathol. Gewerbehyg..

[6] Henson E.V. (1960). The toxicology of some aliphatic alcohols—Part II. J. Occup. Med..

[7] National Institute for Occupational Safety & Health (2000). NIOSH Poket Guide to Chemical Hazards.

[8] Frederick L.J., Schulte P.A., Apol A. (1984). Investigation and control of occupational hazards associated with the use of spirit duplicators. Am. Ind. Hyg. Assoc. J..

[9] Givens M., Kalbfleisch K., Bryson S. (2008). Comparison of methanol exposure routes reported to Texas poison control centers. West. J. Emerg. Med..

[10] Finkelstein Y., Vardi J. (2002). Progressive parkinsonism in a young experimental physicist following long-term exposure to methanol. Neurotoxicology.

[11] Downie A., Khattab T.M., Malik M.I., Samara I.N. (1992). A case of percutaneous industrial methanol toxicity. Occup. Med..

[12] Ryu J., Kim H., Lim K., Ryu D., Le H., Yun J., Kim S., Kim J., Jung-choi K. (2016). Two Cases of Methyl Alcohol Intoxication by Sub-Chronic Inhalation and Dermal Exposure during Aluminum CNC Cutting in a Small-sized subcontracted Factory. Ann. Occup. Environ. Med..

[13] Choi J.-H., Lee S.K., Gil Y.E., Ryu J., Jung-Choi K., Kim H., Yun J.Y. (2017). Neurological Complications Resulting from Non-Oral Occupational Methanol Poisoning. J. Korean Med. Sci..

[14] Solidarity for Workers’ Health (2017). The blind: A Report on Methanol Poisoning Cases in Supply Chains for Samsung and LG Electronics in Korea.

[15] Sakong J., Kang P.S., Kim C.Y., Hwang T.Y., Jeon M.J., Park S.Y., Chung J.H. (2007). Evaluation of reliability of traditional and computerized neurobehavioral tests. Neurotoxicology.

[16] Barceloux D.G., Krenzelok E.P., Olson K., Watson W. (1999). American Academy of Clinical Toxicology Practice Guidelines on the Treatment of Ethylene Glycol Poisoning. Ad Hoc Committee. J. Toxicol. Clin. Toxicol..

[17] Aminian O., Hashemi S., Sadeghniiat-Haghighi K., Shariatzadeh A., Esfahani N.A.H. (2014). Psychomotor effects of mixed organic solvents on rubber workers. Int. J. Occup. Environ. Med..

[18] Bebarta V.S., Heard K., Dart R.C. (2006). Inhalational abuse of methanol products: Elevated methanol and formate levels without vision loss. Am. J. Emerg. Med..

[19] Dutkiewicz B., Kończalik J., Karwacki W. (1980). Skin absorption and per os administration of methanol in men. Int. Arch. Occup. Environ. Health.

[20] Van Liemt G. (2007). Subcontracting in Electronics: From Contract Manufacturers to providers of Electronic Manufacturing Services (EMS). Sectoral Activities Programme, Working Paper.

